# Expression and prognostic significance of Bcl-2 in ovarian tumours.

**DOI:** 10.1038/bjc.1995.509

**Published:** 1995-11

**Authors:** R. Henriksen, E. Wilander, K. Oberg

**Affiliations:** Department of Gynecology and Obstetrics, University Hospital, Uppsala, Sweden.

## Abstract

**Images:**


British Journal of Cancer (1995) 72, 1324-1329

© 1995 Stockton Press All rights reserved 0007-0920/95 $12.00

### Expression and prognostic significance of Bcl-2 in ovarian tumours

Rudi Henriksen[1], Erik Wilander[2] and Kjell Öberg[3]

*Departments of [1]Gynecology and Obstetrics, [2]Pathology and [3]Internal Medicine, University Hospital, S-751 85 Uppsala, Sweden.*

**Summary** The expression of bcl-2 was studied in normal ovaries and in ovarian tumours by immunohistochemical analysis. Normal epithelium was strongly stained in all nine examined ovaries. In comparison, all tumour groups showed a substantially decreased tumour cell expression of the same order of magnitude. Thus, benign tumour cells were weakly stained in two and unstained in two samples, while the remaining eight showed strong expression. Of ten borderline samples, one was unstained and five had weakly and four strongly bcl-2 positive tumour cells. Finally, 24 of 50 malignant tumours showed strong staining, while weak or no expression in tumour cells was found in 16 and 10 samples respectively. The reduced staining deviated significantly from normal ovary for both borderline ($P = 0.02$) and malignant groups ($P = 0.01$). Tumour cell staining with the bcl-2 antibody was significantly reduced when tumour mass had to be left behind compared with those with no visible remaining tumour ($P = 0.03$ and 0.003 for weakly and strongly stained tumours respectively). The expression of bcl-2 in malignant tumour cells was inversely correlated with the expression of p53. Bcl-2 expression was correlated with survival with significantly reduced survival in weakly ($P = 0.02$) and unstained ($P < 0.001$) groups compared with those patients having strongly stained malignant tumour cells. This correlation between the presence of bcl-2 and survival was maintained in the subgroups of patients with advanced disease or with residual tumour bulk and was also the case in patients having p53-positive tumours. Our results indicate an inhibitory role of bcl-2 in development and progression of ovarian tumours.

**Keywords:** bcl-2; p53; ovary; ovarian neoplasms; immunohistochemistry; prognosis

Epithelial ovarian cancer is the leading cause of death in gynaecological malignancy (Petterson, 1991). Treatment is aggressive primary debulking surgery followed by chemotherapy in advanced disease. Although several different clinical trials have been carried out, only a marginal increase in survival has been obtained. Our lack of basic knowledge of the tumour biology underlying this disease presents a major obstacle to improving treatment, as well as to establishing treatment modalities based on aetiological and pathogenetic evidence. Only recently have studies on the role of growth factors in ovarian cancer been carried out, and hitherto two growth factor receptors of prognostic value have been found which appear to be involved in some facet of ovarian tumour development (Slamon *et al.*, 1989; Henriksen *et al.*, 1993).

While much effort has concentrated on examining mechanisms of increased proliferation in cancer development, the regulation of physiological cell death has only recently come into focus. Cell suicide is a well-known fundamental feature in different biological settings (for recent reviews see Raff, 1992; Wyllie, 1993; Kerr and Winterford, 1994). The ultrastructural changes which deviate from the necrotic process were described in 1972 and the process termed apoptosis (Kerr *et al.*, 1972). It provides an efficient mechanism for eliminating cells that are unwanted for some reason and may furthermore be of significance for keeping cell numbers at constant levels in different organs.

Bcl-2 is an oncoprotein, which apparently inhibits apoptosis (McDonnell *et al.*, 1989; Hockenbery *et al.*, 1990). In a few studies on protein expression in various disorders both inhibitory and stimulatory properties towards carcinogenesis were indicated (Castle *et al.*, 1993; Colombel *et al.*, 1993; Leek *et al.*, 1994). In a recent study in non-small-cell lung cancer bcl-2 expression was correlated with survival (Pezella *et al.*, 1993). Thus, in contrast to the teleological viewpoint that decreased apoptosis, which correlates to high bcl-2 expression, should contribute to tumour development by increasing cell mass and decreasing 'cell-cleaning' expression of the anti-apoptotic protein seemed to improve survival.

To further examine its role in tumour development, we have studied the expression of bcl-2 in a variety of ovarian tumours and in normal ovary. In those patients with malignant tumours, we also correlated the expression with survival. Finally, expression was compared with the expression of p53, another parameter of significance for survival in ovarian cancer (Henriksen *et al.*, 1994*a*) and perhaps of significance for the apoptotic process (Yonish-Rouach *et al.*, 1991).

Correspondence: R Henriksen
Received 26 August 1994; revised 1 March 1995; accepted 29 May 1995

### Materials and methods

### *Patient material*

In this prospective study samples were obtained at operation, frozen immediately and kept at −70°C until analysed. Specimens were obtained from 50 malignant epithelial ovarian tumours (details in Table I), ten borderline and 12 benign ovarian tumours and from nine normal ovaries. None of the patients had been subject to treatment before surgery. In most cases total hysterectomy, bilateral salpingoophorectomy and extirpation of the greater omentum was included in the surgical procedure for all stages. Four selected cases with early stage I tumours received no chemotherapy, whereas the others were treated with 4–6 adjuvant cycles of cisplatin and doxorubicin. With few exceptions, patients with stage II–IV tumours underwent 8–10 cycles of cisplatin and doxorubicin as a first line of treatment. Paraplatin or 5-FU and leucovorin were chosen as second-line treatment. Mean follow-up time was 39 months (range 5–60 months); 85% had been followed for more than 2 years. Deaths and censored values in the examined subgroups are shown in the figures.

### *Immunohistochemistry*

Immunohistochemical stainings were performed using 6 mm thick cryosections. Endogenous peroxidase activity was blocked with 0.3% hydrogen peroxide and endogenous avidin-binding activity was blocked using a Blocking kit (Vector Laboratories, Burlingame, CA, USA). After incubation with normal horse serum to block unspecific binding, primary antibody was applied and the sections incubated overnight at 4°C in a humidity chamber. As the primary antibodies, the mouse monoclonal anti-bcl-2 antibody 124

**Table I** Malignant ovarian neoplasms grouped according to histopathological type and FIGO stage

| Type | Stage I | II | III | IV | Total |
|---|---|---|---|---|---|
| Serous | 2 | 1 | 13 | 2 | 18 |
| Mucinous | 5 | 0 | 3 | 1 | 9 |
| Endometrioid | 4 | 2 | 6 | 0 | 12 |
| Clear cell | 5 | 1 | 0 | 0 | 6 |
| Undifferentiated | 0 | 0 | 1 | 0 | 1 |
| Mixed | 2 | 1 | 1 | 0 | 4 |
| Total | 18 | 5 | 24 | 3 | 50 |

diluted 1:200 (Cambridge Research Biochemicals) or the mouse monoclonal anti-p53 antibody PAb 1801 diluted 1:20 were used. After washing in PBS, biotinylated horse anti-mouse Ig (Vector) served as the secondary antibody and the immunoreaction was visualised with a Vectastain Elite ABC kit (Vector) using ethylcarbazole or diaminobenzidine as the chromogen. Finally, the sections were briefly counterstained in Meyer's haematoxylin. All samples had been classified by the same pathologist (EW) using the FIGO classification. Samples were graded as unstained, weakly or strongly cytoplasmic stained with respect to staining of epithelial cells in normal ovaries and tumour cells in the neoplastic groups. Microscopy and evaluation were performed independently by two of the authors. In a few cases with minor disagreement in evaluation, samples were studied together before final classification.

*Statistical analysis*

Survival was measured from the time of primary operation and survival curves constructed by the methods of Kaplan and Meier (1958). Significance was estimated by the log-rank test (Mantel, 1966). Differences in growth factor expression between the groups were estimated with the two-tailed Fisher exact probability test (Armitage, 1987).

### Results

*Expression of bcl-2 in ovarian epithelial cells and in tumour cells in benign borderline and malignant neoplasms*

To examine the significance of bcl-2 in ovarian tumour development we stained several ovarian tumours of varying degrees of malignancy as well as normal ovaries with a monoclonal antibody to bcl-2 protein, which has been used in other studies (Colombel *et al.*, 1993; Pezella *et al.*, 1993; Leek *et al.*, 1994). The results are summarised in Table II. In nine ovaries all epithelial cells stained strongly (Figure 1). In contrast, benign ovarian tumour cells showed no expression in two samples, two were weakly and the remaining eight strongly stained (Figure 2). Of ten borderline tumours one showed no expression and five were weakly and four strongly stained. A corresponding distribution was found in 50 malignant tumours, where 10 tumours did not contain immunoreactive tumour cells, while weak and strong expression were observed in 16 and 24 samples respectively (Figures 3 and 4). Thus, the same pattern of decreased expression in a

**Table II** Positive bcl-2 staining of ovarian epithelial cells and benign or malignant ovarian tumour cells

| | 0 | 1 | 2 |
|---|---|---|---|
| Normal ovary | 0 | 0 | 9 |
| Benign tumours | 2 | 2 | 8 |
| Borderline tumours | 1 | 5 | 4 |
| Malignant tumours | 10 | 16 | 24 |

Expression was graded 0–2 corresponding to no staining, weak or strong staining ($P = 0.02$ and 0.01 for bcl-2 = 0 or bcl-2 = 1 compared with bcl-2 = 2 in the borderline and malignant groups *vs* the normal ovaries).

**Figure 1** Bcl-2 staining of a normal ovary illustrating the strong staining of the normal epithelium.

**Figure 2** Bcl-2 staining of a mucinous ovarian cyst showing an almost negative staining of the benign tumour cells.

**Figure 3** Bcl-2 staining of an ovarian clear cell carcinoma showing a generally strong staining of the malignant tumour cells.

**Figure 4** Bcl-2 staining of an endometrioid highly differentiated ovarian cancer. A varying expression from completely negative to strongly positive malignant tumour cells is seen.

substantial fraction of cases compared with ovarian epithelium was observed in the tumour groups, and the differences were significant for both the borderline ($P = 0.02$) and malignant groups ($P = 0.01$).

Subsequently, tumour staining was compared with the known risk factors stage, residual tumour bulk and differentiation (Table III). While no relation between the presence of bcl-2 and dissemination or differentiation of tumours could be detected, decreased expression was observed significantly more often in patients with residual tumour tissue after primary operation compared with radically operated ones ($P = 0.03$ and $P = 0.003$ for weakly and strongly stained groups respectively).

### *Expression of bcl-2 in the stroma in benign, borderline and malignant neoplasms*

Positive stromal staining was observed in most samples in all groups. Normal ovaries were strongly stained in the cortical parts and only weakly in the core. The tumour groups showed varying staining with no difference between them (results not shown).

### *Comparison of bcl-2 expression in malignant ovarian tumour cells with survival*

The decreased expression of bcl-2 in ovarian neoplasms compared with ovarian epithelium led us to examine its potential correlation with survival. As shown in Figure 5 there was a stepwise and highly significant correlation with survival. Among the group whose tumours stained strongly, 81% were alive at the end of the observation period while for those

**Figure 5** Survival in 50 patients with ovarian cancer, according to expression of bcl-2 in tumour cells. The $P$-value was determined with the log-rank test. Tick marks indicate censored values. Bcl-2 = 0: no staining, Bcl-2 = 1: weak staining, Bcl-2 = 2: strong staining.

**Table III** Expression of bcl-2 related to stage, residual tumour bulk and differentiation

| | *0* | *1* | *2* |
|---|---|---|---|
| Stage I | 2 | 6 | 10 |
| Stage II | 1 | 1 | 3 |
| Stages III-IV | 7 | 9 | 11 |
| No residual tumour bulk | 1 | 9 | 17 |
| Residual tumour bulk | 8 | 7 | 7 |
| Highly differentiated | 3 | 2 | 7 |
| Moderately differentiated | 5 | 6 | 8 |
| Poorly differentiated | 2 | 7 | 4 |

Expression was graded 0-2 corresponding to no staining, weak or strong staining ($P = 0.03$ and $P = 0.003$ for bcl-2 = 1 and 2 respectively in those having residual tumour mass compared with patients macroscopically tumour-free after operation).

with weakly stained and unstained neoplasms, 52% ($P = 0.02$) and 20% ($P < 0.001$) were alive respectively. The difference was even significant between the weakly and unstained groups ($P = 0.03$). This correlation is of the same magnitude as stage (Figure 6), residual tumour bulk (Figure 7) and the new biological prognostic parameter platelet-derived growth factor alpha (PDGF-α) receptor (Henriksen *et al.*, 1993). The strength of bcl-2 as a prognostic marker was tested by relating staining to survival in the subgroups with advanced disease (Figure 6) or residual tumour bulk (Figure 7). For patients in stages III or IV the decreased survival was retained for unstained *vs* strongly stained groups (Figure 6, $P = 0.002$). When patients with residual tumour bulk were stratified for bcl-2 expression in tumour cells significance was found between strongly and unstained groups (Figure 7, $P = 0.03$).

### *Expression of bcl-2 compared with expression of p53 in malignant neoplasms*

The details and significance of p53 expression in ovarian tumours are given in an earlier report (Henriksen *et al.*, 1994*a*). Briefly, almost half of the malignant ovarian tumours stained positive for p53, and positive immunoreactivity was correlated with prognostic variables such as dissemination of disease and residual tumour bulk. Furthermore, positive tumour cell staining correlated with shorter survival in the

**Figure 6** Survival in patients with ovarian cancer in stage I-II or III-IV according to expression of bcl-2 in tumour cells. The $P$-value was determined with the log-rank test. Tick marks indicate censored values. Bcl-2 = 0: no staining, Bcl-2 = 1: weak staining, Bcl-2 = 2: strong staining.

**Figure 7** Survival in patients with ovarian cancer and residual tumour bulk after primary operation according to expression of bcl-2 in tumour cells. The $P$-value was determined with the log-rank test. Tick marks indicate censored values. Bcl-2 = 0: no staining, Bcl-2 = 1: weak staining, Bcl-2 = 2: strong staining.

subgroup of patients with residual tumour bulk. As seen in Table IV an inverse relation between the expression of bcl-2 and p53 was observed. In line with the above observations those patients having p53 positive tumours also positive for bcl-2 experienced significantly better survival compared with the bcl-2-negative counterparts (Figure 8).

### Discussion

Bcl-2 oncoprotein was initially described as a result of the chromosomal translocation t(14;18) observed in a large number of follicular B-cell lines (Tsujimoto *et al*., 1985). The resultant overexpression of bcl-2 often conferred on the affected lymphocytes a resistance to apoptosis (Vaux *et al*., 1988). Later, however, bcl-2 expression was found in normal lymphoid cells and a number of lymphoproliferative disorders without t(14;18) translocation (Pezella *et al*., 1990) and recently, bcl-2 expression was detected in several non-lymphoid tissues (Hockenbery *et al*., 1991).

Ultrastructurally, it was first localised to the inner mitochondrial membranes (Hockenbery *et al*., 1990), but immunoelectron microscopy has demonstrated bcl-2 immunoreactivity to the outer mitochondrial membrane and nuclear envelope and to a lesser degree to the cell membrane (de Jong *et al*., 1994).

The mitochondrial localisation indicated a physiological function mediated via the metabolic functions of this organelle. However, bcl-2 studies on human mutant cell lines that lack mitochondrial DNA suggest that neither apoptosis nor the protective effect of bcl-2 depends on mitochondrial respiration (Jacobsson *et al*., 1993). Other possible functions such as involvement in transmembrane transport have hitherto been purely speculative (de Jong *et al*., 1994), but recent evidence indicates a regulating function of endoplasmic reticulum-associated $Ca^{2+}$ fluxes (Lam *et al*., 1994). Thus, overall, the physiological functions and metabolic pathways remain to be elucidated.

With respect to carcinogenesis the results of the present study on ovaries and ovarian tumours indicate an inhibitory role for bcl-2. Thus, while ovarian epithelium always expressed this oncoprotein, a decreased staining in tumour cells was demonstrated in all tumour groups, which was of the same order of magnitude (Table II). The positive epithelial staining is in line with observations in normal human breast (Hockenbery *et al*., 1991; Leek *et al*., 1994), prostate (Hockenbery *et al*., 1991; Colombel *et al*., 1993) and thyroid gland (Hockenbery *et al*., 1991). In the gastrointestinal tract positive staining was restricted to stem cells and proliferative zones (Hockenbery *et al*., 1991).

**Figure 8** Survival in patients having p53 immunoreactive malignant ovarian tumours according to expression of bcl-2. The *P*-value was determined with the log-rank test. Tick marks indicate censored values. Bcl-2 = 0–1: no or weak staining, Bcl-2 = 2: strong staining.

**Table IV** Expression of bcl-2 compared with p53 in malignant ovarian tumours

| *p53* | *Negative* | *Positive* |
|---|---|---|
| Bcl-2 0–1 | 11 | 15 |
| Bcl-2 2 | 16 | 8 |

Expression was graded 0–2 corresponding to no staining, weak or strong staining ($P = 0.07$ for strongly stained *vs* weakly or negatively stained tumours).

Furthermore, bcl-2 expression correlated significantly with survival such that decreasing survival paralleled the decreased expression in tumour cells (Figure 5). The strength was further evaluated by studying the subgroups of patients with advanced disease or residual tumour bulk, both of which are strong clinical prognostic parameters. In both these subgroups, too, survival was significantly correlated with bcl-2 staining, which underscores an independent role in tumour development and/or progression. In line with our results are the recent observations in non-small-cell lung cancer (Pezella *et al*., 1993) and breast carcinoma (Silvestrini *et al*., 1994), where expression of this oncoprotein correlated with survival. In other recent works the staining in tumour cells was decreased in breast carcinoma compared with normal breast epithelium, but no survival data were reported (Leek *et al*., 1994; Nathan *et al*., 1994). High levels of bcl-2 in cells derived from several cancers resulted in profound growth inhibition while a COOH-terminal deletion mutant of bcl-2 had no effect (Pietenpol *et al*., 1994). In contrast, in human prostate cancers, and especially those refractory to androgen treatment, a stronger bcl-2 staining than corresponding epithelium was noticed and led the authors to suggest a relation to androgen-resistant prostate cancer (Colombel *et al*., 1993). Thus, bcl-2 may serve different functions in the pathobiology of different tissues.

The reason for the correlation of bcl-2 expression with better survival is unknown. According to one theory derived from a study of bcl-2-immunoglobulin transgenic mice (McDonnell *et al*., 1989) bcl-2 may provide a survival advantage to slowly growing tumour cells and thereby decrease the risk of further genetic changes resulting in less aggressive tumours. We have found that proliferation in ovarian cancer estimated by expression of Ki-67 or the S-phase fraction is of strong prognostic significance (Henriksen *et al*., 1994*b*). However, no correlation was observed between the expression of bcl-2 and these proliferation variables (results not shown), and thus the survival advantage of bcl-2 does not seem to depend on a low degree of proliferation.

The human ovarian surface epithelium undergoes cyclic changes of importance for the ovarian function. After ovulation this inconspicuous serosa-like cell layer undergoes rapid proliferation and migrates to cover the site of follicular rupture. However, the regulatory cellular mechanisms are not known in detail.

Also the role and need for apoptotic mechanisms in ovarian epithelial physiology are completely unknown. There is general agreement that epithelial ovarian tumours develop from ovarium epithelium. It is believed that repeated proliferative activity among the epithelial cells predispose to genetic damage and to malignant conversion. Apoptosis might be an effective antineoplastic mechanism by eliminating damaged or transformed cells. Therefore, loss of function should be expected to act in a tumorigenic way. However, our results and those of others (Pezella *et al*., 1993; Silvestrini *et al*., 1994) with bcl-2 staining indicate the opposite effect with decreased survival correlating with decreased expression in tumour cells. Furthermore, staining of normal ovaries and benign and borderline tumours revealed reduced expression in a number of both tumour groups, indicating that this oncoprotein expression may be depressed at an early stage of tumour development. Whether this plays an early pathogenic role is unknown. Does decreased bcl-2 staining define a group of benign tumours with a particular propensity to progress to malignancy? Support for such a subgroup is found in our recent report showing expression of Ki-67 in a few benign ovarian tumours, which suggest proliferative activity in a subgroup of benign ovarian tumours (Henriksen *et al.*, 1994*b*). Our knowledge of ovarian tumours is mainly restricted to histopathology. In general not much is known of the biological aspects and, in particular, time-related changes are unknown.

Recently, bcl-2 was shown to prevent p53-induced apoptosis at the permissive temperature in rodent cells transformed with E1A plus a p53 temperature-sensitive mutant (Chiou *et al.*, 1994). While bcl-2 diverted the activity of wild-type p53 from apoptosis to induction of growth arrest, it did not affect the localisation or the levels of p53 indicating an effect downstream of the tumour-suppressor gene product. In other recent reports the same group presented evidence for a p53-inducible decrease of bcl-2 and increase of bax expression (Miyashita *et al.*, 1994*a*; Selvakumaran *et al.*, 1994) and for a p53-dependent negative response element in the *bcl-2* gene (Miyashita *et al.*, 1994*b*). This might be expected to result in an inverse expression of p53 and bcl-2, and this has been reported in normal tissues (Pezella *et al.*, 1994) as well as in mammarian tumours (Silvestrini *et al.*, 1994) and in the present work, too, an inverse correlation was found. However, while an interaction with bcl-2 function is supposed to exist with the wild-type form of p53, not much is known of a corresponding function with the different mutational forms of the protein. In one report overexpression of mutant p53 could induce down-regulation of bcl-2 both at mRNA and protein level (Haldar *et al.*, 1993). Whether this is valid in our material remains to be elucidated.

The number of patients in our study does not allow a multivariate analysis. To elucidate the possible significance of bcl-2 independently of p53, the correlation with survival in the p53-positive group was examined. As seen in Figure 8 a significant correlation between bcl-2 expression and survival was still detectable which underlines that the observed correlation between bcl-2 expression and survival is not secondary to mutational inactivation of p53. In the group negatively stained for p53 the material was too small to make similar statistical estimations.

In conclusion, we have reported a strong epithelial staining of bcl-2 in ovaries and a reduced tumour cell immunoreactivity in benign, borderline and malignant epithelial ovarian tumours. Overall, the expression in malignant tumours strongly correlated with enhanced survival, which was also observed in subgroups of patients with advanced disease or residual tumour bulk as well as in patients having p53-positive tumours, indicating an independent role in ovarian carcinogenesis.

**Acknowledgements**
This work was supported by grants from the Swedish Cancer Research Foundation (RMC), Project No. 1925-B91-06XAC, 1759, Swedish Medical Research Council, Lions Cancer Foundation and Erik, Karin and Gösta Selanders Foundation. The skilled technical assistance of Ms Rajni Dyal is gratefully acknowledged.

### References

ARMITAGE BG. (1987). Statistical methods in medical research. Second edn. Blackwell: Oxford.

CASTLE VP, HEIDELBERGER KP, BROMBERG J, OU X, DOLE M AND NUNEZ G. (1993). Expression of the apoptosis-suppressing protein bcl-2, in neuroblastoma is associated with unfavorable histology and N-myc amplification. *Am. J. Pathol.*, **143**, 1543–1550.

CHIOU S-K., RAO L AND WHITE E. (1994). Bcl-2 blocks p53-dependent apoptosis. *Mol. Cell. Biol.*, **14**, 2556–2563.

COLOMBEL M, SYMMANS F, GIL S, O'TOOLE KM, CHOPIN D, BENSON M, OLSSON CA, KORSMEYER S AND BUTTYAN R. (1993). Detection of the apoptosis-suppressing oncoprotein bcl-2 in hormone-refractory human prostate cancers. *Am. J. Pathol.*, **143**, 390–400.

DE JONG D, PRINS FA, MASON DY, REED JC, VAN OMMEN GB AND KLUIN PM. (1994). Subcellular localization of the bcl-2 protein in malignant and normal lymphoid cells. *Cancer Res.*, **54**, 256–260.

HALDAR S, NEGRINI M, MONNE M, SABBIONI S AND CROCE CM. (1994). Down-regulation of bcl-2 by p53 in breast cancer cells. *Cancer Res.*, **43**, 2095–2097.

HENRIKSEN R, FUNA K, WILANDER E, BÄCKSTRÖM T, RIDDERHEIM M AND ÖBERG K. (1993). Expression and prognostic significance of platelet-derived growth factor and its receptors in epithelial ovarian neoplasms. *Cancer Res.*, **53**, 4550–4554.

HENRIKSEN R, STRANG P, WILANDER E, BÄCKSTRÖM T, TRIBUKAIT B AND ÖBERG K. (1994*a*). p53 expression in epithelial ovarian neoplasms: Relationship to clinical and pathological parameters, Ki-67 expression and flow cytometry. *Gynecol. Oncol.*, **53**, 301–306.

HENRIKSEN R, STRANG P, BÄCKSTRÖM T, WILANDER E, TRIBUKAIT B AND ÖBERG K. (1994*b*). Ki-67 immunostaining and DNA flow cytometry as prognostic factors in epithelial ovarian cancers. *Anticancer Res.*, **14**, 603–608.

HOCKENBERY D, NUNEZ G, MILLIMAN C, SCHREIBER RD AND KORSMEYER SJ. (1990). Bcl-2 is an inner mitochrondial membrane protein that blocks programmed cell death. *Nature*, **348**, 334–336.

HOCKENBERY DM, ZUTTER M, HICKEY W, NAHM M AND KORSMEYER SJ. (1991). Bcl-2 protein is topographically restricted in tissues characterized by apoptotic cell death. *Proc. Natl Acad. Sci. USA*, **88**, 6961–6965.

JACOBSSON MD, BURNE JF, KING MP, MIYASHITA T, REED JC AND RAFF MC. (1993). Bcl-2 blocks apoptosis in cells lacking mitochondrial DNA. *Nature*, **361**, 365–369.

KAPLAN EL AND MEIER P. (1958). Nonparametric estimation from incomplete observations. *J. Am. Stat. Ass.*, **53**, 457–481.

KERR JFR AND WINTERFORD CM. (1994). Apoptosis. *Cancer*, **73**, 2013–2026.

KERR JF, WYLLIE AH AND CURRIE AR. (1972). Apoptosis: a basic biologic phenomenon with wide-ranging implications in tissue kinetics. *Br. J. Cancer*, **26**, 239–257.

LAM M, DUBYAK G, CHEN L, NUNEZ G, MIESFELD RL AND DISTELHORST CW. (1994). Evidence that bcl-2 repress apoptosis by regulating endoplasmic reticulum-associated $Ca^{++}$ fluxes. *Proc. Natl Acad. Sci. USA*, **91**, 6569–6573.

LEEK RD, KAKLAMANIS L, PEZELLA F, GATTER KC AND HARRIS AL. (1994). Bcl-2 in normal human breast and carcinoma, association with oestrogen receptor-positive, epidermal growth factor receptor-negative tumors and *in situ* cancer. *Br. J. Cancer*, **69**, 135–139.

MCDONNELL TJ, DEANE N, PLATT FM, NUNEZ G, JAEGER U, MCKEARN JP AND KORSMEYER SJ. (1989). Bcl-2 immunoglobulin transgenic mice demonstrate extended B cell survival and follicular lymphoproliferation. *Cell*, **57**, 79–88.

MANTEL N. (1966). Evaluation of survival data and two new rank order statistics arising in its consideration. *Cancer Chemother. Rep.*, **50**, 163–170.

MIYASHITA T, KRAJEWSKI S, KRAJEWSKA M, WANG HG, LIN HK, LIEBERMAN DA, HOFFMAN B AND REED JC. (1994*a*). Tumor suppressor p53 is a regulator of bcl-2 and bax gene expression *in vitro* and *in vivo*. *Oncogene*, **9**, 1799–1805.

MIYASHITA T, HARIGAI M, HANADA HM AND REED JC. (1994*b*). Identification of a p53-dependent negative response element in the bcl-2 gene. *Cancer Res.*, **54**, 3131–3135.

NATHAN B, GUSTERSON B, JADAYEL S, O'HARE M, ANBAZHAGAN R, JAYATILAKE H, EBBS S, MICKLEM K, PRICE K, GELBER R, REED R, SENN H-J, GOLDHIRSCH A AND DYER MJS. (1994). Expression of bcl-2 on primary breast cancer and its correlation with tumour phenotype. *Ann. Oncol.*, **5**, 409–414.

PETTERSON F. (ed.) (1991). Annual report on the results of treatment in gynecologic cancer. *Int. J. Gynecol. Obstet.*, **21**, (suppl 36).

PEZELLA F, TSE AGD, CORDELL JL, PULFORD KAF, GATTER KC AND MASON DY. (1990). Expression of the bcl-2 oncogene protein is not specific for the 14;18 chromosal translocation. *Am. J. Pathol.*, **137**, 225–232.

PEZELLA F, TURLEY H, KUZU LI, TUNGEKAR MF, DUNNILL MS, PIERCE CB, HARRIS A, GATTER KC AND MASON DY. (1993). Bcl-2 protein in non-small cell lung carcinoma. *New. Engl. J. Med.*, **329**, 690–694.

PEZELLA F, MICKLEM K, TURLEY H, PULFORD K, KOCIALKOWSKI S, DELIA D, AIELLO A, BICKNELL R, SMITH K, HARRIS AL, GATTER KC AND MASON DY. (1994). Antibody for detecting p53 protein by immunohistochemistry in normal tissues. *J. Clin. Pathol.*, **47**, 592–596.

PIETENPOL JA, PAPADOPOULOS N, MARKOWITZ S, WILLSON JKV, KINZLER KW AND VOGELSTEIN B. (1994). Paradoxical inhibition of solid tumor cell growth by bcl2. *Cancer Res.*, **54**, 3714–3717.

RAFF MC. (1992). Social controls on cell survival and cell death. *Nature*, **356**, 397–400.

SELVAKUMARAN M, LIN H-K, MIYASHITA T, WANG HG, KRAJEWSKI S, REED JC, HOFFMAN B AND LIEBERMANN D. (1994). Immediate early up-regulation of bax expression by p53 but not TGF-β1: a paradigm for distinct apoptotic pathways. *Oncogene*, **9**, 1791–1798.

SILVESTRINI R, VENERONI S, DAIDONE MG, BENINI E, BORACCHI P, MEZETTI M, DI FRONZO G., RILKE F AND VERONESI U. (1994). The bcl-2 protein; a prognostic indicator strongly related to p53 protein in lymph node-negative breast cancer patients. *J. Natl Cancer Inst.*, **86**, 499–504.

SLAMON DJ, GODOLPHIN W, JONES LA, HOLT JA, WONG SE, KEITH DE, LEVIN WJ, STUART SG, UDOVE J, ULLRICH A AND PRESS MF. (1989). Studies of the HER-/neu proto-oncogene in human breast and ovarian cancer. *Science*, **244**, 707–712.

TSUJIMOTO Y, GORHAM J, COSSMAN J, JAFFE E AND CROCE CM. (1985). The t(14:18) chromosome translocations involved in β-cell neoplasms results from mistakes in VDJ joining. *Science*, **299**, 1390–1393.

VAUX DL, CORY S AND ADAMS JM. (1988). Bcl-2 promotes haemopoitic cell survival and cooperates with c-myc to immortalize pro-B cells. *Nature*, **335**, 440–442.

WYLLIE AH. (1993). Apoptosis (The 1992 Frank Rose Memorial Lecture). *Br. J. Cancer*, **67**, 205–208.

YONISH-ROUACH E, RESNITZKY D, LOTEM J, SACHS L, KIMCHI A AND OREN M. (1991). Wild-type p53 induces apoptosis of myeloid leukaemic cells that is inhibited by interleukin 6. *Nature*, **352**, 345–347.


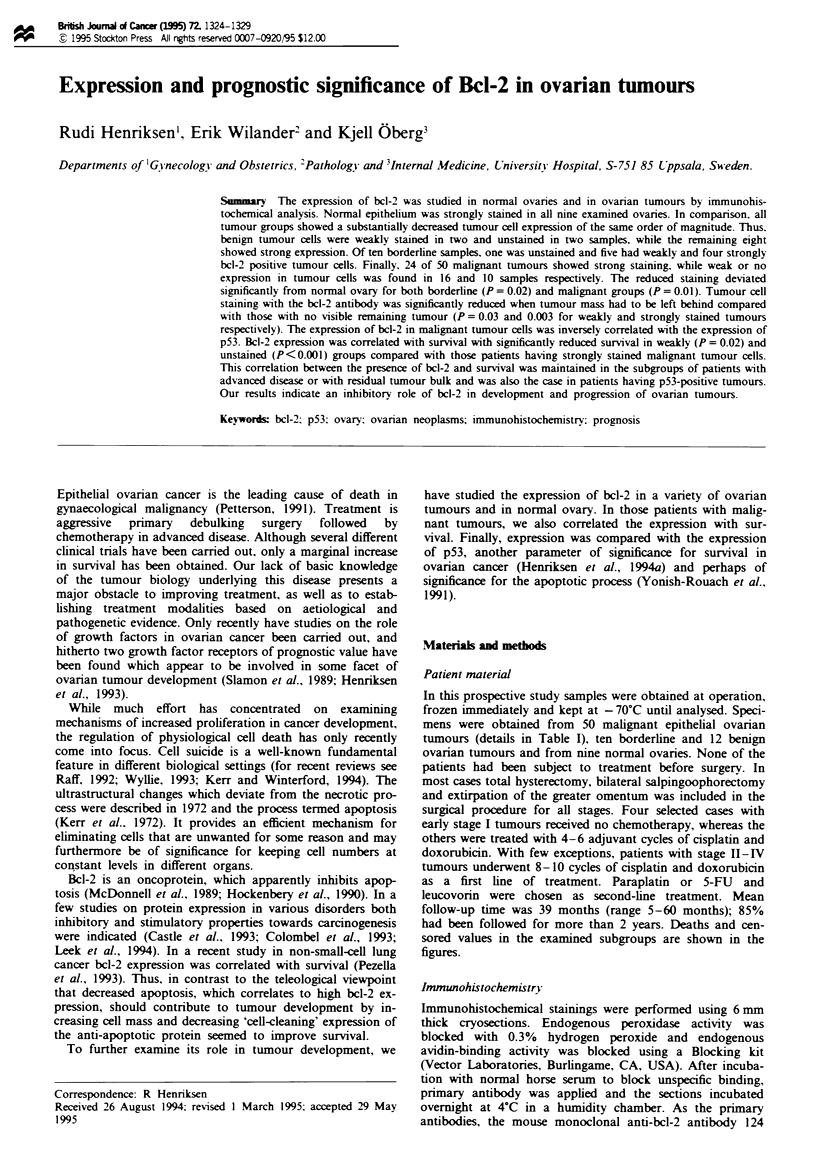

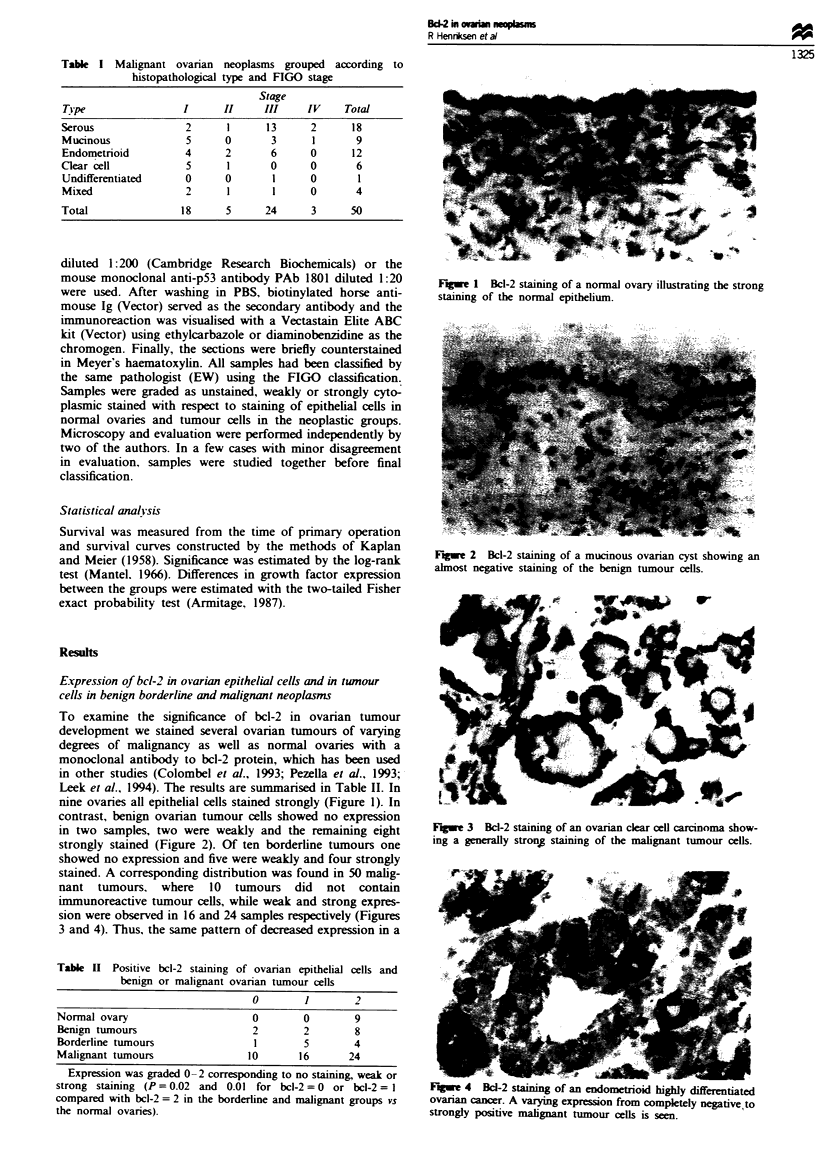

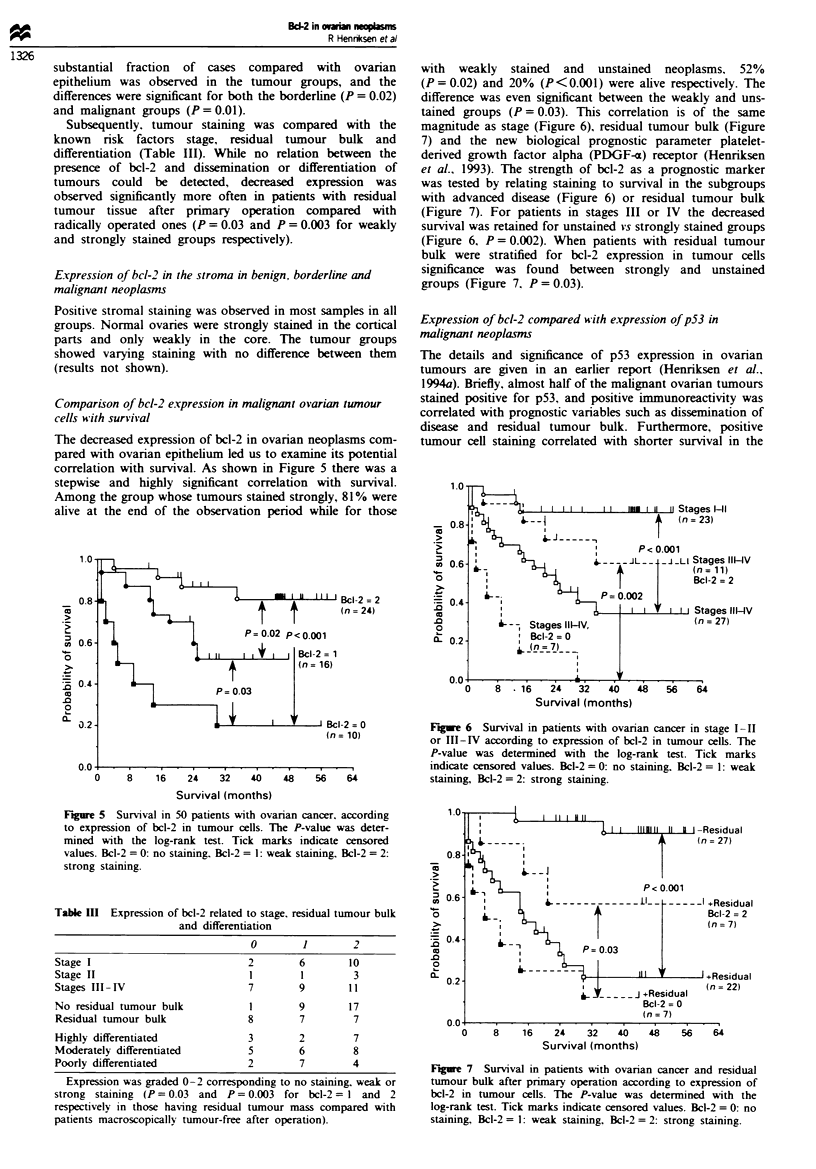

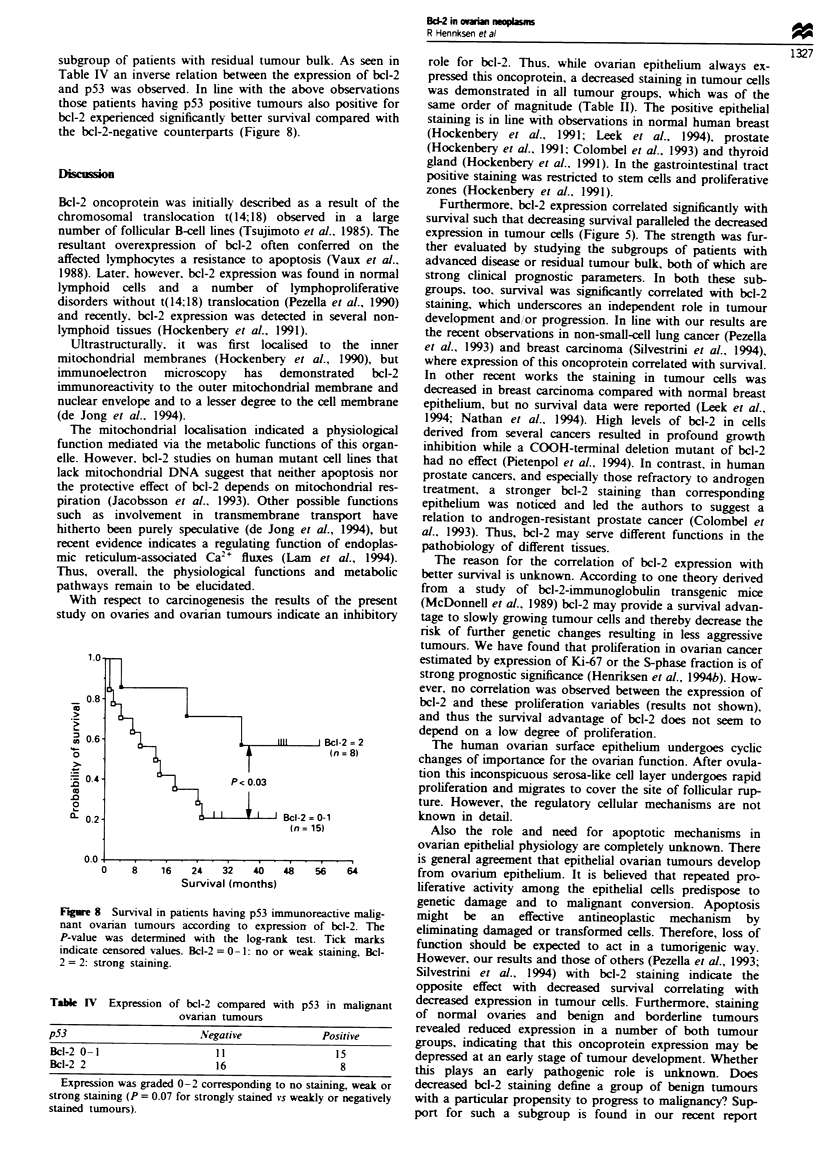

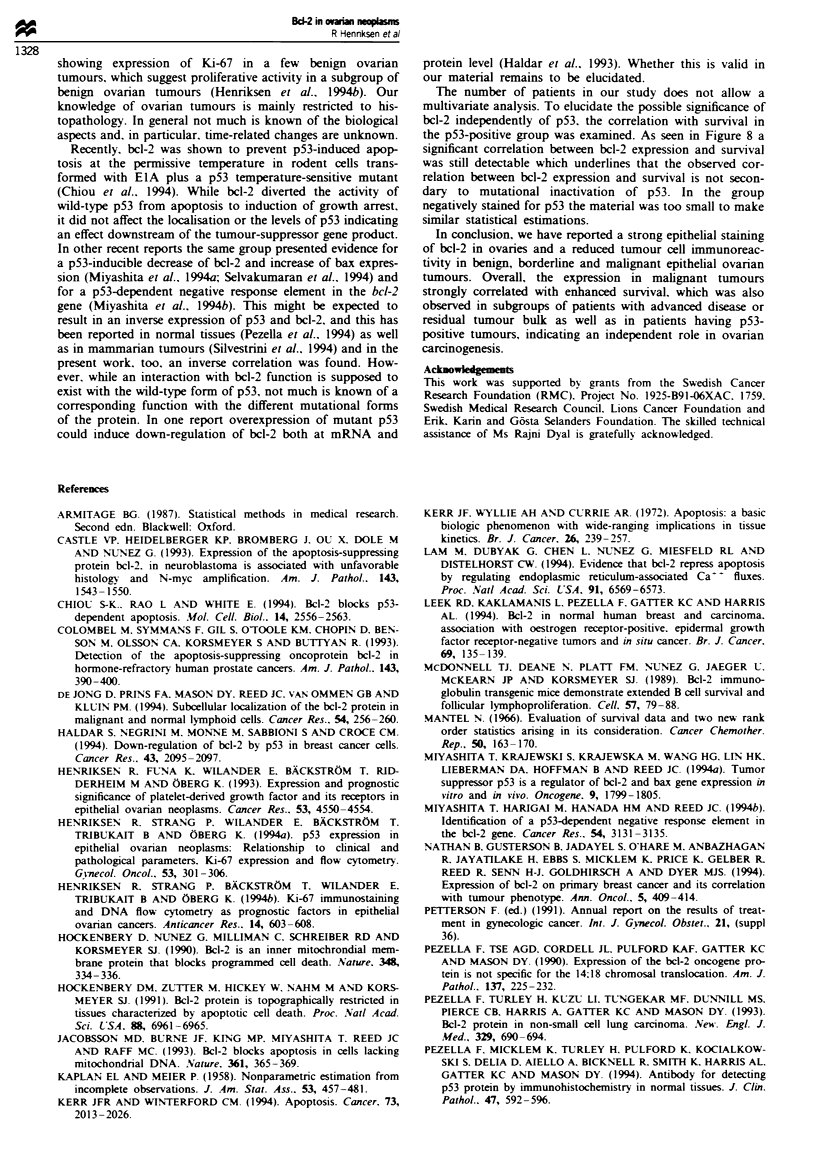

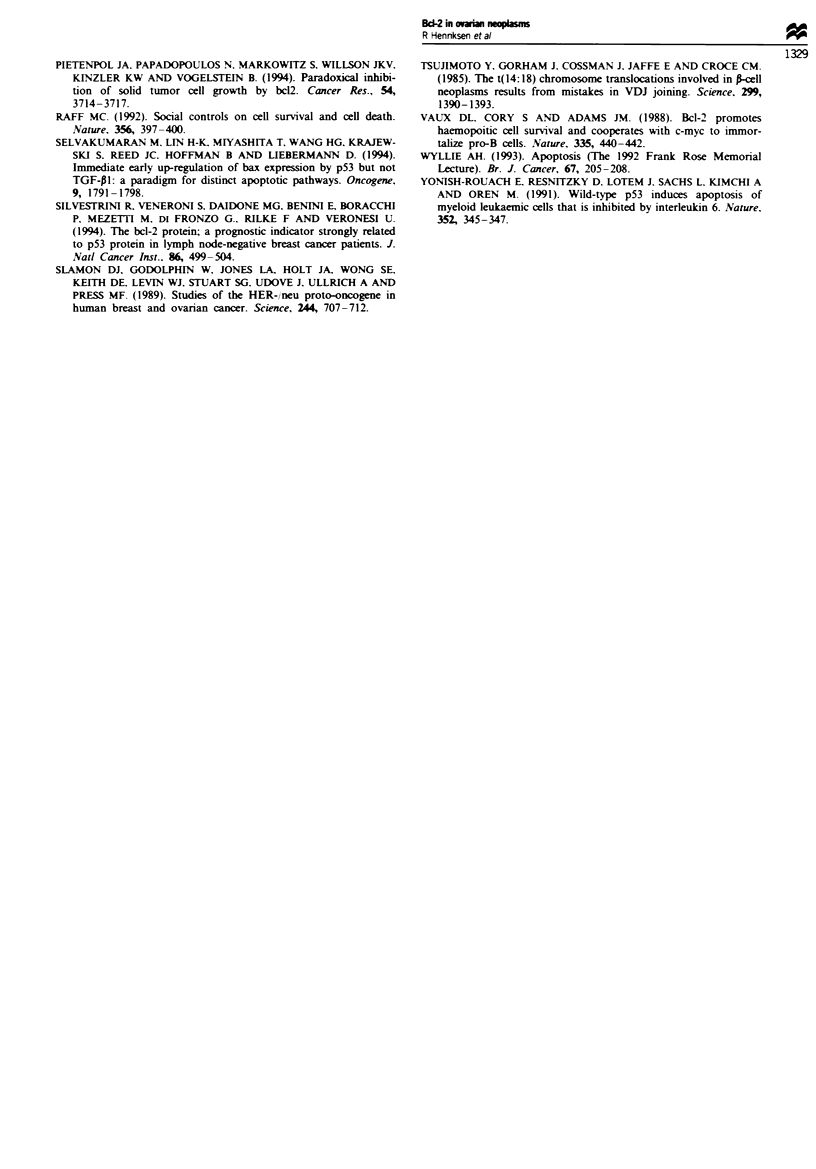


## References

[OCR_00625] Castle V. P., Heidelberger K. P., Bromberg J., Ou X., Dole M., Nuñez G. (1993). Expression of the apoptosis-suppressing protein bcl-2, in neuroblastoma is associated with unfavorable histology and N-myc amplification.. Am J Pathol.

[OCR_00632] Chiou S. K., Rao L., White E. (1994). Bcl-2 blocks p53-dependent apoptosis.. Mol Cell Biol.

[OCR_00634] Colombel M., Symmans F., Gil S., O'Toole K. M., Chopin D., Benson M., Olsson C. A., Korsmeyer S., Buttyan R. (1993). Detection of the apoptosis-suppressing oncoprotein bc1-2 in hormone-refractory human prostate cancers.. Am J Pathol.

[OCR_00647] Haldar S., Negrini M., Monne M., Sabbioni S., Croce C. M. (1994). Down-regulation of bcl-2 by p53 in breast cancer cells.. Cancer Res.

[OCR_00653] Henriksen R., Funa K., Wilander E., Bäckström T., Ridderheim M., Oberg K. (1993). Expression and prognostic significance of platelet-derived growth factor and its receptors in epithelial ovarian neoplasms.. Cancer Res.

[OCR_00663] Henriksen R., Strang P., Backstrom T., Wilander E., Tribukait B., Oberg K. (1994). Ki-67 immunostaining and DNA flow cytometry as prognostic factors in epithelial ovarian cancers.. Anticancer Res.

[OCR_00656] Henriksen R., Strang P., Wilander E., Bäckström T., Tribukait B., Oberg K. (1994). p53 expression in epithelial ovarian neoplasms: relationship to clinical and pathological parameters, Ki-67 expression and flow cytometry.. Gynecol Oncol.

[OCR_00677] Hockenbery D. M., Zutter M., Hickey W., Nahm M., Korsmeyer S. J. (1991). BCL2 protein is topographically restricted in tissues characterized by apoptotic cell death.. Proc Natl Acad Sci U S A.

[OCR_00671] Hockenbery D., Nuñez G., Milliman C., Schreiber R. D., Korsmeyer S. J. (1990). Bcl-2 is an inner mitochondrial membrane protein that blocks programmed cell death.. Nature.

[OCR_00683] Jacobson M. D., Burne J. F., King M. P., Miyashita T., Reed J. C., Raff M. C. (1993). Bcl-2 blocks apoptosis in cells lacking mitochondrial DNA.. Nature.

[OCR_00692] Kerr J. F., Winterford C. M., Harmon B. V. (1994). Apoptosis. Its significance in cancer and cancer therapy.. Cancer.

[OCR_00696] Kerr J. F., Wyllie A. H., Currie A. R. (1972). Apoptosis: a basic biological phenomenon with wide-ranging implications in tissue kinetics.. Br J Cancer.

[OCR_00702] Lam M., Dubyak G., Chen L., Nuñez G., Miesfeld R. L., Distelhorst C. W. (1994). Evidence that BCL-2 represses apoptosis by regulating endoplasmic reticulum-associated Ca2+ fluxes.. Proc Natl Acad Sci U S A.

[OCR_00707] Leek R. D., Kaklamanis L., Pezzella F., Gatter K. C., Harris A. L. (1994). bcl-2 in normal human breast and carcinoma, association with oestrogen receptor-positive, epidermal growth factor receptor-negative tumours and in situ cancer.. Br J Cancer.

[OCR_00718] Mantel N. (1966). Evaluation of survival data and two new rank order statistics arising in its consideration.. Cancer Chemother Rep.

[OCR_00715] McDonnell T. J., Deane N., Platt F. M., Nunez G., Jaeger U., McKearn J. P., Korsmeyer S. J. (1989). bcl-2-immunoglobulin transgenic mice demonstrate extended B cell survival and follicular lymphoproliferation.. Cell.

[OCR_00731] Miyashita T., Harigai M., Hanada M., Reed J. C. (1994). Identification of a p53-dependent negative response element in the bcl-2 gene.. Cancer Res.

[OCR_00725] Miyashita T., Krajewski S., Krajewska M., Wang H. G., Lin H. K., Liebermann D. A., Hoffman B., Reed J. C. (1994). Tumor suppressor p53 is a regulator of bcl-2 and bax gene expression in vitro and in vivo.. Oncogene.

[OCR_00736] Nathan B., Gusterson B., Jadayel D., O'Hare M., Anbazhagan R., Jayatilake H., Ebbs S., Micklem K., Price K., Gelber R. (1994). Expression of BCL-2 in primary breast cancer and its correlation with tumour phenotype. For the International (Ludwig) Breast Cancer Study Group.. Ann Oncol.

[OCR_00761] Pezzella F., Micklem K., Turley H., Pulford K., Jones M., Kocialkowski S., Delia D., Aiello A., Bicknell R., Smith K. (1994). Antibody for detecting p53 protein by immunohistochemistry in normal tissues.. J Clin Pathol.

[OCR_00749] Pezzella F., Tse A. G., Cordell J. L., Pulford K. A., Gatter K. C., Mason D. Y. (1990). Expression of the bcl-2 oncogene protein is not specific for the 14;18 chromosomal translocation.. Am J Pathol.

[OCR_00755] Pezzella F., Turley H., Kuzu I., Tungekar M. F., Dunnill M. S., Pierce C. B., Harris A., Gatter K. C., Mason D. Y. (1993). bcl-2 protein in non-small-cell lung carcinoma.. N Engl J Med.

[OCR_00774] Pietenpol J. A., Papadopoulos N., Markowitz S., Willson J. K., Kinzler K. W., Vogelstein B. (1994). Paradoxical inhibition of solid tumor cell growth by bcl2.. Cancer Res.

[OCR_00779] Raff M. C. (1992). Social controls on cell survival and cell death.. Nature.

[OCR_00781] Selvakumaran M., Lin H. K., Miyashita T., Wang H. G., Krajewski S., Reed J. C., Hoffman B., Liebermann D. (1994). Immediate early up-regulation of bax expression by p53 but not TGF beta 1: a paradigm for distinct apoptotic pathways.. Oncogene.

[OCR_00791] Silvestrini R., Veneroni S., Daidone M. G., Benini E., Boracchi P., Mezzetti M., Di Fronzo G., Rilke F., Veronesi U. (1994). The Bcl-2 protein: a prognostic indicator strongly related to p53 protein in lymph node-negative breast cancer patients.. J Natl Cancer Inst.

[OCR_00798] Slamon D. J., Godolphin W., Jones L. A., Holt J. A., Wong S. G., Keith D. E., Levin W. J., Stuart S. G., Udove J., Ullrich A. (1989). Studies of the HER-2/neu proto-oncogene in human breast and ovarian cancer.. Science.

[OCR_00803] Tsujimoto Y., Gorham J., Cossman J., Jaffe E., Croce C. M. (1985). The t(14;18) chromosome translocations involved in B-cell neoplasms result from mistakes in VDJ joining.. Science.

[OCR_00807] Vaux D. L., Cory S., Adams J. M. (1988). Bcl-2 gene promotes haemopoietic cell survival and cooperates with c-myc to immortalize pre-B cells.. Nature.

[OCR_00812] Wyllie A. H. (1993). Apoptosis (the 1992 Frank Rose Memorial Lecture).. Br J Cancer.

[OCR_00816] Yonish-Rouach E., Resnitzky D., Lotem J., Sachs L., Kimchi A., Oren M. (1991). Wild-type p53 induces apoptosis of myeloid leukaemic cells that is inhibited by interleukin-6.. Nature.

[OCR_00644] de Jong D., Prins F. A., Mason D. Y., Reed J. C., van Ommen G. B., Kluin P. M. (1994). Subcellular localization of the bcl-2 protein in malignant and normal lymphoid cells.. Cancer Res.

